# Activation of ERBB4 in Glioblastoma Can Contribute to Increased Tumorigenicity and Influence Therapeutic Response

**DOI:** 10.3390/cancers10080243

**Published:** 2018-07-25

**Authors:** Jacqueline F. Donoghue, Lauren T. Kerr, Naomi W. Alexander, Sameer A. Greenall, Anthony B. Longano, Nicholas G. Gottardo, Rong Wang, Viviane Tabar, Timothy E. Adams, Paul S. Mischel, Terrance G. Johns

**Affiliations:** 1 Oncogenic Signalling Group, Hudson Institute of Medical Research, 21–37 Wright Street, Clayton, VIC 3168, Australia; jacqueline.donoghue@unimelb.edu.au (J.F.D.); Lauren.Kerr@cancer.org.uk (L.T.K.); sameer.greenall@hudson.org.au (S.A.G.); 2 Department of Molecular and Translational Science, Monash University, Clayton, VIC 3168, Australia; 3 Telethon Kids Cancer Centre, Telethon Kids Institute, University of Western Australia, Perth, WA 6008, Australia; naomi.alexander@telethonkids.org.au (N.W.A.); Nicholas.Gottardo@telethonkids.org.au (N.G.G.); 4 Department of Anatomical Pathology, Monash Medical Centre, Clayton, VIC 3168, Australia; anthony.longano@monashhealth.org; 5 Department of Neurosurgery and Center for Stem Cell Biology, Memorial Sloan Kettering Cancer Center, New York, NY 10065, USA; wangr@mskcc.org (R.W.); tabarv@mskcc.org (V.T.); 6 Biomedical Manufacturing, Commonwealth Scientific and Industrial Research Organisation (CSIRO), Parkville, VIC 3052, Australia; Tim.Adams@csiro.au; 7 Ludwig Institute for Cancer Research, University of California San Diego, La Jolla, CA 92093, USA; pmischel@ucsd.edu

**Keywords:** GBM, EGFR, ERBB4, prognosis, therapy

## Abstract

Glioblastoma (GBM) is often resistant to conventional and targeted therapeutics. ErbB2 Receptor Tyrosine Kinase 4 (ERBB4) is expressed throughout normal brain and is an oncogene in several pediatric brain cancers; therefore, we investigated ERBB4 as a prognostic marker and therapeutic target in GBM. Using RT-qPCR, we quantified mRNA encoding total ERBB4 and known ERBB4 variants in GBM and non-neoplastic normal brain (NNB) samples. Using immunohistochemistry, we characterized the localization of total and phosphorylated ERBB4 (p-ERBB4) and EGFR protein in archived GBM samples and assessed their association with patient survival. Furthermore, we evaluated the effect of ERBB4 phosphorylation on angiogenesis and tumorigenicity in GBM xenograft models. Total *ERBB4* mRNA was significantly lower in GBM than NNB samples, with the juxtamembrane JM-a and cytoplasmic CYT-2 variants predominating. ERBB4 protein was ubiquitously expressed in GBM but was not associated with patient survival. However, high p-ERBB4 in 11% of archived GBM samples, independent of p-EGFR, was associated with shorter patient survival (12.0 ± 3.2 months) than was no p-ERBB4 (22.5 ± 9.5 months). Increased ERBB4 activation was also associated with increased proliferation, angiogenesis, tumorigenicity and reduced sensitivity to anti-EGFR treatment in xenograft models. Despite low *ERBB4* mRNA in GBM, the functional effects of increased ERBB4 activation identify ERBB4 as a potential prognostic and therapeutic target.

## 1. Introduction

Glioblastoma (GBM) is the most aggressive primary brain cancer in adults [1], and about 13,000 cases arise annually in the USA [2]. GBM tumors are diffuse [3] and highly invasive, with prominent therapeutic resistance [4,5], resulting in high morbidity and mortality (median survival, <1 year). Currently, amplification/overexpression of epidermal growth factor receptor (EGFR), which occurs in ~52% of patients with primary GBM [6,7,8,9], is the strongest predictor of shorter overall survival [6], and most new therapeutics are targeted to EGFR. Limited prognostic and targeted therapeutic options are available for the remaining patients.

Analysis of other EGFR family members—ERBB2 (HER2), ERBB3 and ERBB4—indicates that ERBB4 is the second most prominent member in GBM [10]. ERBB4 is present in neuron-like elements in GBM [11] and in some GBM cell lines [12]. ERBB4 also plays a critical role in normal neural development [13] and is localized in normal adult neural cells in the frontal cortex, hippocampus and cerebellum [14,15]. It is highly expressed in pediatric brain cancers such as medulloblastoma [16] and ependymoma [17]. Therefore, evaluating ERBB4 as a prognostic marker and therapeutic target for GBM is warranted.

**Figure 1:**
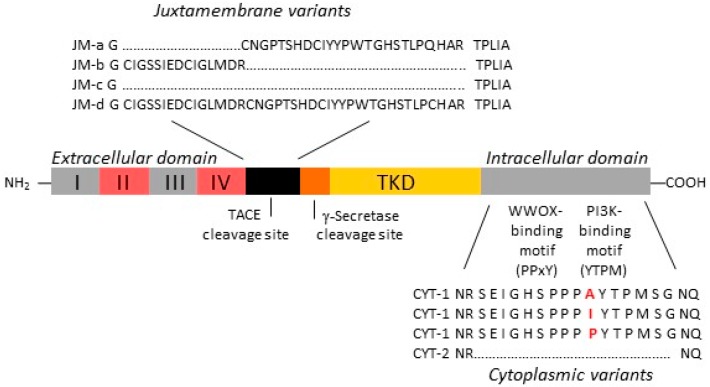
Schematic representation of ERBB4 juxtamembrane (JM-a,b,c,d) and cytoplasmic (CYT-1,2) variant sequences. JM-a- and JM-d-containing variants are cleaved by tumour necrosis factor converting enzyme (TACE), releasing the extracellular domain. Further cleavage occurs at the γ-secretase site of the JM-a- and JM-d- containing variants, releasing a soluble intracellular fragment containing the tyrosine kinase domain (TKD) and the cytoplasmic tail from the inner cell membrane. JM-b and JM-c have no TACE cleavage site; therefore, variants containing either of these remain as a full-length receptor. CYT-1-containing variants include a 16 amino acid sequence that is absent from CYT-2-containing variants and contains WW domain-containing oxidoreductase (WWOX)- and PI3K-binding motifs. Amino acid sequences were generated from NCBI Nucleotide database.

Like EGFR, ERBB4 is a transmembrane receptor tyrosine kinase (RTK), with a ligand-binding extracellular domain and an intracellular catalytic kinase domain [18]. Alternative RNA splicing can result in six variants: Tumor necrosis factor-alpha converting enzyme (TACE)-cleavable extracellular domains (JM-a, JM-d), non-cleavable extracellular domains (JM-b, JM-c) and intracellular domains (CYT-1, CYT-2) (Figure 1). These variants are expressed in a tissue-specific manner, with JM-b predominating in the brain, particularly the cerebellum [19]. In medulloblastoma, all ERBB4 variants are expressed, with JM-a [16] and CYT-1 [20] predominating.

The ERBB4 variants have divergent functions. CYT-1 expression in breast cancer cell lines, including MCF7, induces ligand-dependent growth inhibition and differentiation that reflect tumor-suppressor behavior [21,22]. Conversely, transfecting MCF7 with JM-a–CYT-2 enhances anchorage-independent growth and proliferation [19]. In addition, transfecting NR6 cells with JM-a-CYT-2 results in increased proliferation and colony formation, however there is no increase in cells transfected with Jm-b-CYT-2 [23]. JM-a–CYT-2’s oncogenic activity results from constitutive cleavage (no TACE requirement), constitutive phosphorylation (i.e., activation; no ligand requirement) [24,25,26] and increased auto-kinase activity [24,27]. Furthermore, *ERBB4* somatic mutations identified in melanoma increase ERBB4’s kinase activity and capacity to transform cell lines [28]. Therefore, although ERBB4 is often associated with tumor-suppressive behavior, it has a strong oncogenic capacity that is tissue- and variant-specific.

ERBB4 function in GBM has not been fully evaluated. Because better prognostic markers and alternative therapeutic options are needed for GBM patients, we characterized ERBB4 protein expression and activation in a cohort of 53 GBM samples with matching survival data, as well as variant expression in 28 GBM samples. We also assessed ERBB4′s tumorigenic activity in an in vivo GBM model.

## 2. Results

### 2.1. Increased Oncogenic ERBB4 Variant Expression Is Associated with GBM

**Figure 2:**
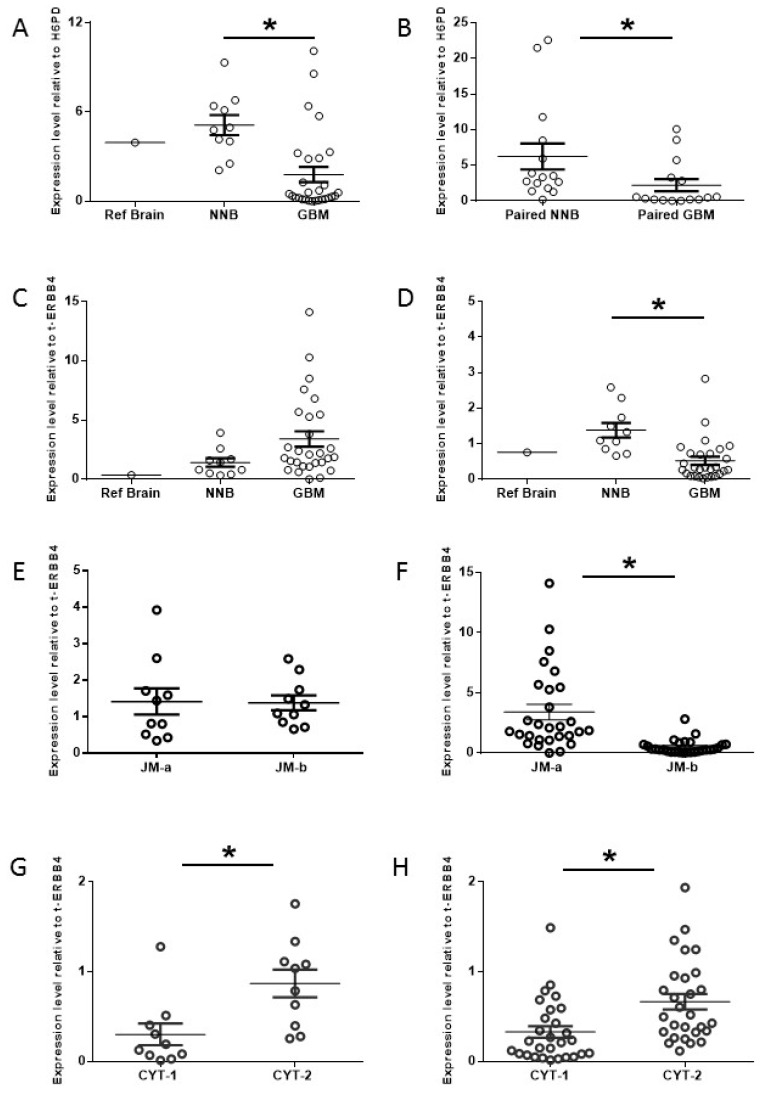
Total ERBB4 and ERBB4 variant expression. ERBB4 expression in 10 non-neoplastic normal brain (NNB), 28 glioblastoma (GBM), and 15 paired NNB and GBM samples. Expression levels were determined by RT-qPCR; total ERBB4 levels were standardized to *H6PD* mRNA, and variant levels were standardized to total *ERBB4* mRNA levels. All measurements were made simultaneously but are displayed separately to aid comparison. Reference brain (Ref Brain) was a pooled brain sample from 7 donors (Ambion, Thermo-Fisher, Waltham, MA, USA) used for comparison. Total *ERBB4* mRNA expression in Ref Brain, NNB and GBM samples (**A**). Total *ERBB4* mRNA expression in paired NNB and GBM samples (**B**). Expression of JM-a (**C**) and JM-b (**D**) variants of ERBB4 in Ref Brain, NNB and GBM samples. Comparison of JM-a and JM-b expression levels in NNB (**E**) and GBM (**F**). (Note that the data shown in (**E**,**F**) are rescaled from (**C**) and (**D**).) CYT-1 and CYT-2 expression levels in NNB (**G**) and GBM (**H**). Experiments were performed in triplicate (* *p* < 0.05, Student’s *t*-test).

To determine whether ERBB4 is expressed in GBM, we measured total ERBB4 expression in GBM samples derived from patients diagnosed with World Health Organization WHO grade III or IV primary glioma, NNB and Ref Brain. Total *ERBB4* mRNA levels were significantly lower in GBM than in NNB samples (*p* = 0.002), although a subset of GBM patients, 4/28 (14%), exhibited higher *ERBB4* mRNA levels than the mean for NNB samples (Figure 2A). An analysis of the TCGA data for 100 sequential GBM patients showed that a similar proportion, 15%, had high *ERBB4* mRNA levels (Figure S1A). We then compared total *ERBB4* mRNA levels in 15 GBM samples paired with NNB biopsied from the contralateral hemisphere and found significantly lower *ERBB4* mRNA levels in GBM than in paired NNB (*p* = 0.04) (Figure 2B), confirming reports that an overall reduction in *ERBB4* mRNA is associated with GBM [10,11,29].

Analysis of ErBB4 expression is complicated by the possibility of at least six different splice variants [30]. We therefore evaluated the *ERBB4* expression levels of the four juxta-membrane variants (JM-a,b,c,d) and the two cytoplasmic variants (CYT-1,2) (Figure 1) relative to total ERBB4 in 28 GBM and 10 NNB samples by using RT-qPCR. More JM-a mRNA (cleavable variant) was present in GBM samples than in NNB, although this difference was not significant (*p* = 0.09) (Figure 2C). Conversely, significantly less JM-b mRNA (non-cleavable variant) was found in GBM than in NNB (*p* = 0.004) (Figure 2D), indicating a trend toward preferential JM-a expression (over JM-b) in GBM. The JM-a and JM-b mRNA levels were similar in NNB samples (Figure 2E), whereas significantly more JM-a than JM-b was observed in GBM samples (*p* = 0.001) (Figure 2F), reflecting a significant increase in the cleavable ERBB4 variant in GBM. Additionally, the JM-c (non-cleavable) and JM-d (cleavable) mRNA levels were significantly lower than the JM-a and JM-b mRNA levels found in GBM and NNB; however, the JM-c and JM-d mRNA levels were significantly higher in GBM than NNB (*p* = 0.04 and *p* = 0.04, respectively) (Figure S2A,B), indicating that an increase in rare ERBB4 variants is associated with GBM. Therefore, although total *ERBB4* mRNA levels are significantly lower in GBM than in NNB, there is a significant upregulation in cleavable ERBB4 (JM-a) compared with non-cleavable ERBB4 (JM-b) in GBM.

CYT-1 and CYT-2 mRNA levels did not differ significantly between GBM and NNB (*p* = 0.45, Figure S2C; *p* = 0.22, Figure S2D, respectively). However, significantly more CYT-2 than CYT-1 was expressed in NNB (*p* = 0.004; Figure 2G) and GBM (*p* = 0.006; Figure 2H), indicating that CYT-2 is the predominant cytoplasmic *ERBB4* variant in the brain (NNB and GBM). This is consistent with an earlier report showing that CYT-2 is the predominant transcript in neural tissues [31]. A similar pattern was observed in GBM in the TCGA data set (Figure S1B), thereby supporting our finding. Thus, GBM is associated with lower levels of total ERBB4 but higher levels of cleavable ERBB4 (JM-a) in association with CYT-2.

### 2.2. ERBB4 Activation in GBM Is Associated with Shorter Overall Survival

IHC has been used to characterize EGFR protein expression as a prognostic indicator for GBM [6,8] and to predict which patients are likely to benefit from EGFR-targeted therapy [32]. Despite ERBB4 protein being observed in GBM, ERBB4’s role in GBM tumorigenesis and patient survival has not been characterized. Using IHC, the expression patterns of ERBB4 and p-ERBB4, along with EGFR and p-EGFR, were assessed to identify whether these are associated with patient survival.

**Figure 3:**
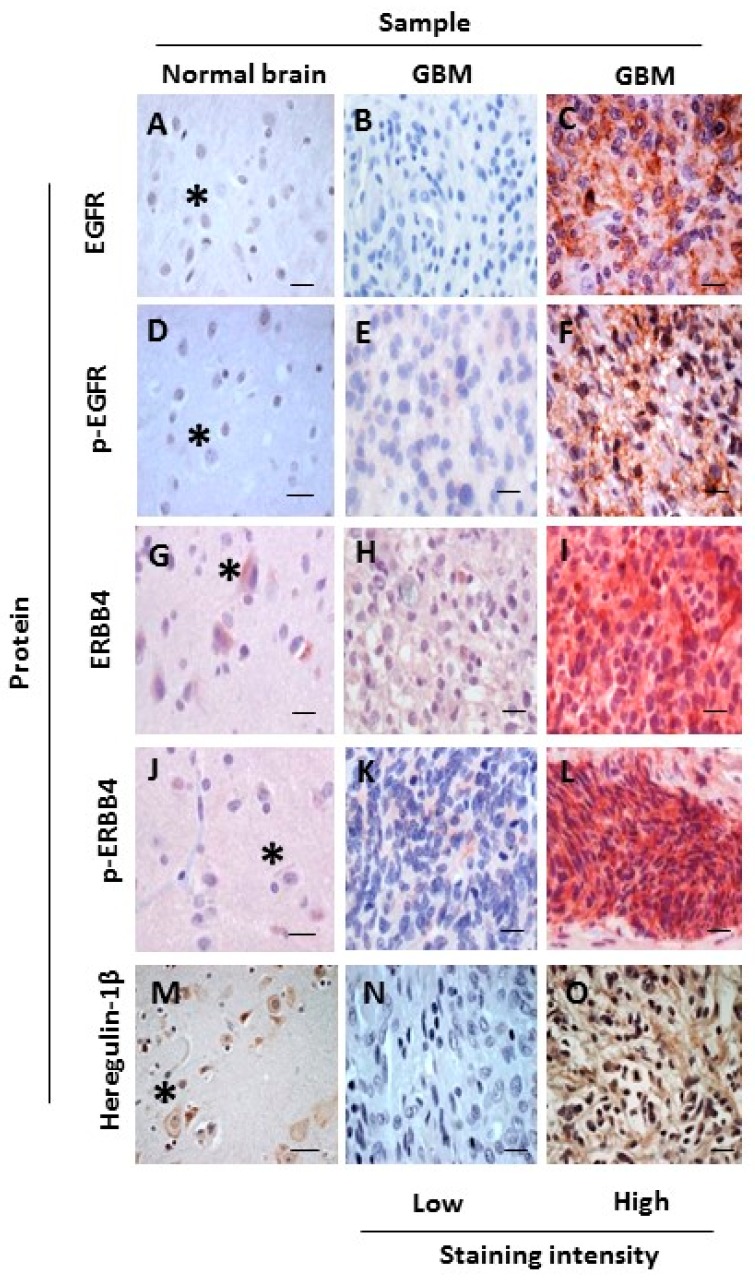
Representative IHC images of occipital regions in an NNB sample and a GBM sample. Samples were stained for EGFR (**A**–**C**), p-EGFR (**D**–**F**), ERBB4 (**G**–**I**), p-ERBB4 (**J**–**L**) and heregulin-1β protein (**M**–**O**). Images are representative of high and low staining intensity of ERBB proteins (stained with VECTOR^®^ Red Alkaline Phosphatase (red), Burlingame, CA, USA) and of heregulin-1β protein (stained with 3,3′-Diaminobenzidine (DAB) (brown); nuclei were counterstained with hematoxylin (blue). Asterisks denote neurons. Scale bars, 25 µm.

Using serial sections, we initially evaluated EGFR and p-EGFR expression in NNB and GBM. EGFR (Figure 3A) and p-EGFR (Figure 3D) protein were not observed in NNB (0/10), as previously reported [33]. By contrast, EGFR was observed, with variable intensities, in 85% of GBM samples (45/53) (Figure 3B,C), and p-EGFR was observed, with diffuse and often patchy staining, in 77% of GBM samples (41/53) (Figure 3E,F). Therefore, EGFR expression and activation are upregulated in GBM.

Next, we investigated whether ERBB4, p-ERBB4, and the ERBB3- and ERBB4-specific ligand heregulin-1β are present in NNB and GBM. Numerous neuronal cells were ERBB4- and heregulin-1β-positive, and these were diffusely distributed in all NNB samples (10/10) (Figure 3G,M, respectively). In GBM samples (53/53), ERBB4-positive (Figure 3H,I), p-ERBB4-positive (Figure 3K,L) and heregulin-1β-positive (Figure 3N,O) cells were more densely grouped and had variable staining intensity. Commercial p-ERBB4-specific antibody showed some cross-reactivity with p-EGFR by western blotting; therefore, to accurately assess p-ERBB4, we compared consecutive serial sections independently stained for p-EGFR and p-ERBB4 and removed areas that were p-EGFR-positive from the p-ERBB4 analysis (Figure S3A–D). We rarely observed p-ERBB4 staining in NNB samples (1/10; 10%) (Figure 3J), whereas p-ERBB4 was present in 89% of GBM samples (47/53) (Figure 3K,L). Therefore, heregulin-1β and ERBB4 protein are endogenously expressed in NNB and GBM, consistent with the detection of total ERBB4 mRNA expression in both sample types. Furthermore, increased heregulin-1β and ERBB4 expression in GBM is associated with increased ERBB4 activation, consistent with relatively higher mRNA expression of the oncogenic JM-a ERBB4 isoform in GBM.

**Figure 4:**
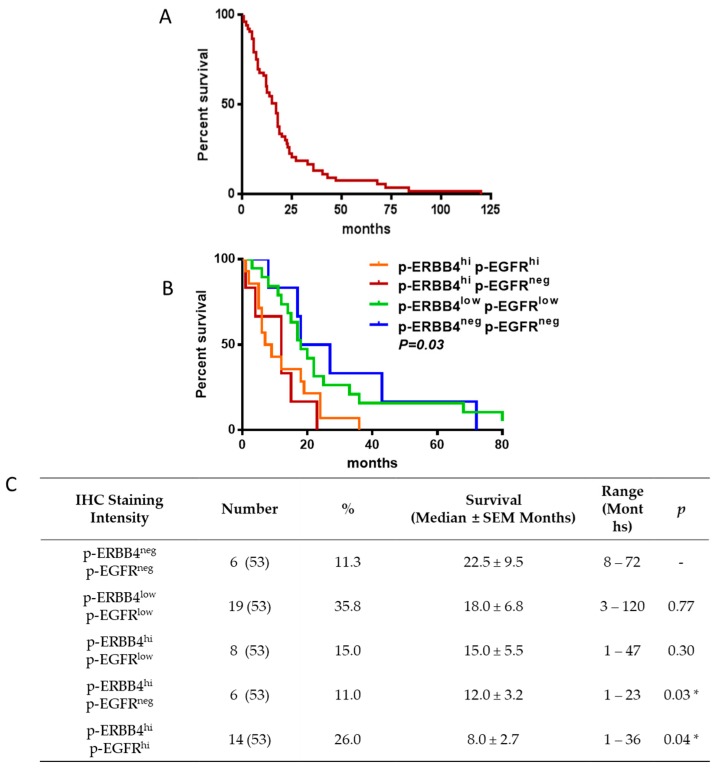
Comparative survival of patients with GBM. The overall survival for the 53 patients with GBM for whom survival data were available ranged from 1 to 120 months (**A**). Graphical (**B**) and tabulated (**C**) survival outcomes associated with patient samples with high (hi), low (low) and no (neg) p-ERBB4 or p-EGFR staining by IHC. Combinations not shown in the table were not identified. (* *p* < 0.05 vs. p-ERBB4^neg^ p-EGFR^neg^ samples, Student’s *t*-test).

Next, we determined whether the extent of ERBB4 and/or EGFR phosphorylation is a prognostic marker(s) in GBM. The 53 GBM patients whose samples were analyzed by IHC had a median overall survival time ± s.e.m. of 17.0 ± 3.1 months (range, 1–120) from diagnosis (Figure 4A). To characterize p-ERBB4 and p-EGFR staining, the samples were divided into three groups based on the intensity of p-ERBB4 and p-EGFR staining: none (neg), low (low) and high (hi). Patients whose tumors were p-ERBB4-negative and p-EGFR-negative had a mean survival of 22.5 ± 9.5 months (Figure 4B,C). As the p-ERBB4 staining intensity increased, patient survival significantly decreased: 12.0 ± 3.2 months for p-ERBB4^hi^ p-EGFR^neg^, and 8.0 ± 2.7 months for p-ERBB4^hi^ p-EGFR^hi^ (*p* = 0.03 and *p* = 0.04, respectively, vs. p-ERBB4^neg^ p-EGFR^neg^). Heregulin-1β staining intensity did not correlate with patient survival. Taken together, these data indicate that increased p-ERBB4, either independently of or together with increased p-EGFR, is associated with significantly shorter patient survival, suggesting that increased p-ERBB4 may be a prognostic marker for GBM. These data also reveal p-ERBB4 as a potential therapeutic target in GBM. 

### 2.3. ERBB4 Activation Enhances GBM Cell Growth and Tumorigenicity

**Figure 5:**
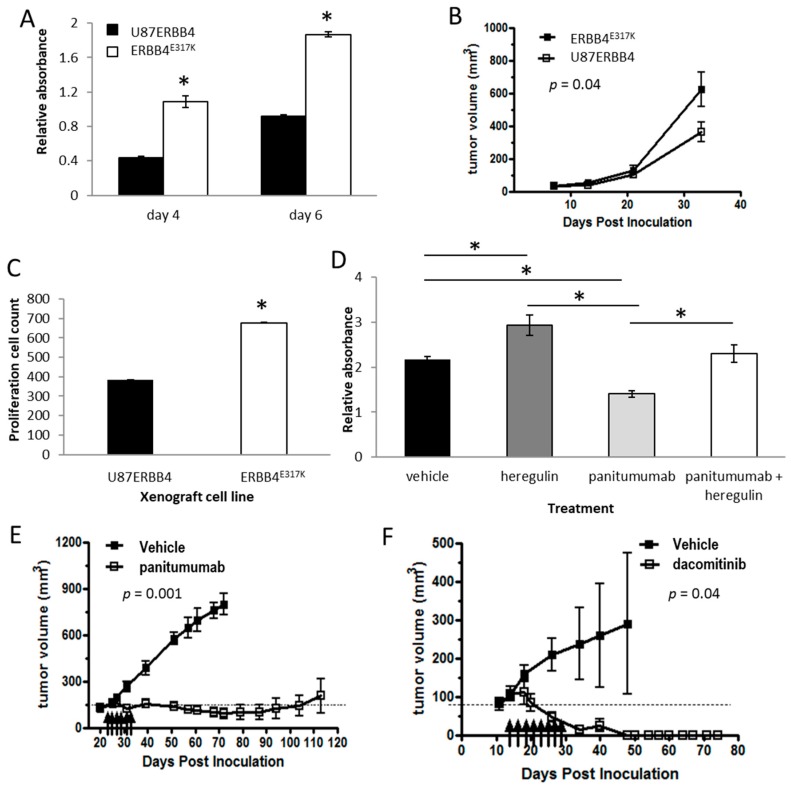
Activated ERBB4 and cell growth potential. The proliferation of U87MG cells infected with retrovirus carrying wild-type ERBB4 (U87ERBB4) was compared with hyper-activated ERBB4-expressing cells (ERBB4^E317K^) in vitro using an MTS proliferation assay at day 4 and 6 following drug addition (**A**), in vivo using xenograft tumor growth measured over time (**B**) and ex vivo using IHC analysis of Ki67 staining (**C**). MTS proliferation assay of SF767 cells treated with heregulin-1β (40 ng/mL), panitumumab (10 µg/mL) or both (**D**). Tumor volumes of SF767 xenografts treated with panitumumab (**E**) or dacomitinib (**F**). Arrows denote treatment times. Bars indicate mean values ± SEM (*n* = 4). Asterisks indicate bars that are significantly different from each other (* *p* < 0.05, Student’s *t*-test). All in vitro experiments were performed in triplicate. The in vivo tumor data include six repeats per group.

To study the function of activated ERBB4 in GBM, U87ERBB4 and ERBB4^E317K^ cell proliferation and tumor growth were compared. These retrovirally-infected cells express wild-type (U87ERBB4) or constitutively active (ERBB4^E317K^) versions of the ErbB4 JM-a CYT-2 isoform. The ERBB4^E317K^ mutant exhibits increased basal activation, and has been shown to induce transformation in NIH3T3 cells [28]. ERBB4^E317K^ cells were significantly more proliferative in vitro (*p* = 0.001), as determined by MTS assay (Figure 5A), and showed significantly more p-ERK signaling (*p* = 0.01), as determined by Bio-Plex assay (Figure S4A). No significant change in p-AKT signaling occurred in response to constitutively activated ERBB4 (*p* = 0.67) (Figure S4B). Additionally, ERBB4^E317K^ xenografts were significantly larger than U87ERBB4 xenografts (*p* = 0.04) (Figure 5B) and were more proliferative than U87ERBB4 cells, as demonstrated by Ki67 staining (*p* = 0.001) (Figure 5C). Therefore, constitutively activated ERBB4 increases both proliferation and tumorigenicity through increased activation of ERK signaling in GBM cells.

Several GBM clinical trials have targeted EGFR but with limited long-term benefit [34]. We speculated that EGFR and ERBB4 interaction may contribute to this limited efficacy by providing an alternative signaling pathway. To test this, we investigated the effect of anti-EGFR therapy on SF767 cells. Whilst the use of such cell lines may not be an entirely accurate representation of in vivo cancer cell behavior, these cells provided a good model for studying the effect of such inhibition as they express both endogenously activated EGFR and ERBB4 [12]. SF767 cells treated with heregulin-1β proliferated significantly more in vitro than did untreated cells, as determined by MTS assay, demonstrating that ERBB4 in these cells is ligand-responsive (Figure 5D). Treatment with panitumumab, an anti-EGFR monoclonal antibody, significantly reduced cell growth in vitro, demonstrating these cells were responsive to EGFR inhibition. However, simultaneous panitumumab and heregulin-1β treatment significantly increased cell growth in vitro compared with panitumumab-only treatment (*p* = 0.04). Therefore, inhibiting EGFR activity in the presence of hyper-activated ERBB4 (endogenous activation plus exogenous ligand) can significantly increase GBM cell growth. Furthermore, inhibiting EGFR with panitumumab increased p-ERK activity (Figure S5A), as determined by Bio-Plex assay and confirmed by western blotting (Figure S5B), but had minimal effect on p-AKT activity (Figure S5C), consistent with signaling downstream of the JM-a-CYT-2 isoform, which does not contain a PI3K-binding motif. We then tested the addition of an ErbB4-Fc ligand trap to these cells to specifically antagonize ErbB4-driven signaling [35], and found that this inhibited cell proliferation (Figure S6). Together, these data demonstrate that constitutive activation of ERBB4 (through mutation or ligand stimulation) promotes GBM cell growth and that, following anti-EGFR treatment, ERBB4 activation may result in increased tumor growth by upregulating ERK activity.

### 2.4. Endogenous ERBB4 Activation Permits Tumor Survival During Anti-EGFR Therapy

Because ERBB4 activation results in increased GBM growth and attenuates anti-EGFR therapy in vitro, we next evaluated the capacity of endogenous ERBB4 activation to maintain GBM cell growth during anti-EGFR therapy in vivo. We compared the growth of SF767 cells, as subcutaneous xenografts, following panitumumab (anti-EGFR) or dacomitinib (pan-ERBB inhibitor) treatment. Following panitumumab treatment, the tumors were significantly smaller than those in vehicle-treated mice (indicating no upfront resistance to panitumumab), and tumor growth was static for >60 days after tumor initiation, after which time all tumors started to regrow (Figure 5E). Dacomitinib treatment resulted in complete tumor disappearance, with no tumors visible 60 days after tumor initiation (Figure 5F). Therefore, SF767 cells are dependent on ERBB signaling. Additionally, during anti-EGFR therapy, GBM cells expressing other ERBB family members, such as ERBB4, may tolerate anti-EGFR therapy, allowing tumors to regrow later.

### 2.5. ERBB4 Has a Vascular Role in GBM

**Figure 6:**
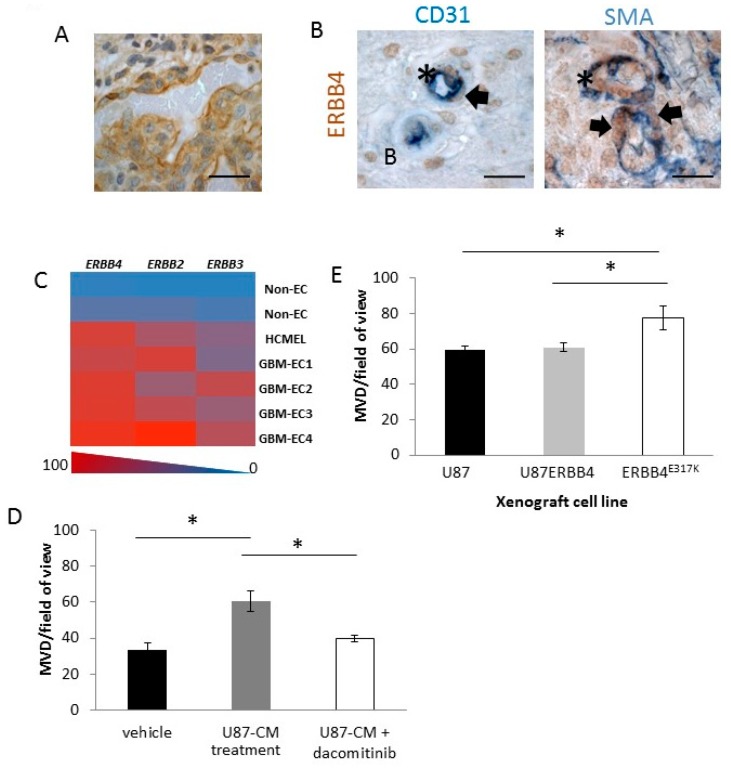
Angiogenic role of ERBB4. (**A**,**B**) p-ERBB4 expression on the vasculature of the GBM glomeruloid structure identified with DAB (brown), with nuclei counterstained with hematoxylin (**A**). IHC double stains demonstrated p-ERBB4 (brown) expression on endothelial cells (CD31, blue) and on pericytes (SMA, blue) in GBM patient samples (**B**). All IHC was performed on four biological replicates. Arrows point to pericytes, and asterisks identify endothelial cells. Scale bars, 25 µm. (**C**) Heat map of *ERBB4*, *ERBB3* and *ERBB2* mRNA expression of endothelial cells isolated from four samples of GBM (GBM-EC), one normal brain endothelial cell line (HCMEC) and two non-endothelial cell lines (non-EC), as measured by RT-qPCR. RT-qPCR was performed on triplicate samples. (**D**,**E**) MVD counts from subcutaneous agar plugs containing U87MG conditioned medium (U87-CM) following treatment with dacomitinib (**D**); representative images are shown in Figure S9A. MVD counts for U87MG, U87ERBB4 and ERBB4^E317K^ xenografts (**E**); representative images are shown in Figure S9B. Bars indicate mean values ± SEM (*n* = 4). Asterisks indicate bars that are significantly different from each other (*p* < 0.05, one-way ANOVA).

During our initial IHC investigations, we noted p-ERBB4 localized on GBM vasculature, including the enlarged hyper-proliferative endothelial cell structures that are prominent GBM features (Figure 6A), and we noted ERBB4 and heregulin-1β, but not EGFR, localization (Figure S7A–D). ERBB4 was co-localized on endothelial cells (CD31^+^) and pericytes (SMA^+^) in the GBM vasculature (Figure 6B), whereas GBM-derived endothelial cells expressed mainly ERBB4 and to a lesser extent ERBB2 and ERBB3 (Figure 6C). These data indicate that ERBB4 contributes to vascularization in GBM.

As vascular ERBB4 expression in GBM could make ERBB4 a viable target for anti-angiogenic therapy, we evaluated whether ERBB4-positive vessels in a tumor microenvironment can be regulated by anti-ERBB therapy, by using a subcutaneous alginate plug model of angiogenesis (Figure S8A–D). BALB/c mice received alginate plugs containing PBS or serum-free conditioned medium from U87MG cells (U87-CM), to mimic the soluble factors secreted by GBM cells, plus daily intraperitoneal administration of PBS or the pan-ERBB inhibitor dacomitinib. After 7 days, samples were collected for analysis. The U87-CM-containing plugs had a significantly higher microvascular density (MVD) than the PBS-containing alginate plugs (*p* = 0.04) (Figure 6D), indicating that U87-CM contained angiogenic stimulants. The MVD of U87-CM-containing plugs from dacomitinib-treated mice was significantly lower than that of the U87-CM-containing plugs from PBS-treated mice (*p* = 0.03) (Figure 6D), indicating that the angiogenic stimulation induced by U87-CM was reduced by inhibiting ERBBs. Furthermore, ERBB4^E317K^ xenografts had a significantly higher tumor MVD than U87MG or U87ERBB4 xenografts (*p* = 0.03) (Figure 6E), indicating that increased ERBB4 activation contributes to vascularization. Overall, ERBB4 expression and activation in the GBM tumor or the surrounding vessels contributes significantly to GBM microvessel growth and stability, making ERBB4 a possible anti-angiogenic target.

## 3. Discussion

Our GBM samples, while demonstrating lower overall levels of ERBB4 mRNA in comparison to normal matched brain samples, show elevated JM-a and CYT-2 expression, suggesting that JM-a–CYT-2 has a role in GBM tumorigenesis, similar to its oncogenic role in breast cancer [24] but distinct from medulloblastoma (in which JM-a–CYT-1 is the oncogenic variant) [16,20]. Indeed, the shorter survival of patients whose tumors displayed p-ERBB4, and the enhanced tumor growth driven by constitutively active ERBB4, strongly supports an oncogenic role for ERBB4. The mechanism that leads to activation is unknown, but the ERBB4 ligand, heregulin-1β, was present in most samples.

ERBB4’s oncogenic activity results partly from JM-a cleavage from the membrane and CYT nuclear transfer, which drives transcription and increases proliferation [19,24] or hyperplasia [36]. CYT-2 signals through the MAPK pathway [31,37], whereas CYT-1 preferentially signals through PI3K [20] because of its PI3K-binding motif (YTPM). CYT-1 also contains three PPxY motifs [38] (where x is alanine, proline or isoleucine), which provide binding sites for the E3-ligases NEDD4 and ITCH, ensuring that CYT-1 is more efficiently ubiquitinated and degraded than CYT-2 [39,40]. Furthermore, in a recent study showing that EGFR can dimerize with CYT-1 or CYT-2, only CYT-2 protected EGFR from ligand-induced ubiquitination and degradation, by sequestering EGFR and thus preventing its binding to the E3-ligase c-CBL and the adaptor GRB2 [41]. Additionally, increased CYT-2 expression resulted in increased EGFR expression. These findings strongly support an oncogenic role for ERBB4 and indicate that JM-a–CYT-2 expression in GBM may protect EGFR from degradation, which could then result in resistance to EGFR-targeted therapeutics.

Furthermore, increased p-ERK signaling during anti-EGFR treatment or due to increased ERBB4 hyper-activation, as observed here, demonstrates that GBM cells have a high degree of dynamic and adaptable signaling activity. Such plasticity in signaling necessitates flexible treatment: that is, drug combinations that inhibit multiple signaling pathways and prevent pathway switching due to robust oncogenic receptor activity should be used.

An increase in activated ERBB4 was also associated with shorter patient survival, independently of EGFR activation. *ERBB4* is not mutated in GBM [42], and the observed increase in ERBB4 activation was almost certainly due to the robust heregulin-1β expression observed. The population in which this occurred was relatively small, thus limiting multi-variant analysis with respect to other prognostic indicators (such as *MGMT* and *IDH1*, the latter of which is not likely to contain mutations in our sample set); however, the p-ERBB4 increase may still be clinically significant, as it could contribute to the failure of EGFR-targeted therapeutics.

Numerous clinical trials of EGFR-targeted monotherapy have not significantly extended patient survival [43,44]. Therapeutic resistance to EGFR inhibitors can result from intrinsic compensatory mechanisms such as *KRAS* mutations [45] or from acquired resistance due to tumor heterogeneity and/or receptor cross-talk and heterodimerization [12,29,46]. In this study, heregulin-1β enabled GBM cells to continue to proliferate even in the presence of an EGFR-targeted therapeutic. In addition, a specific ErbB4 antagonist in the form of an ErbB4 ligand trap, inhibited the growth of SF767 cells, suggesting that an ErbB4 autocrine system at least partially drives the growth of these cells. It is therefore possible that GBM-derived heregulin-1β [11] (or other ERBB4 ligands, such as HB-EGF [47]) may promote resistance to EGFR-targeted therapies in a subset of GBM patients through compensatory ERBB4 signaling. Furthermore, in our xenograft model, EGFR-targeted monotherapy failed to prevent tumor recurrence, whereas the pan-ERBB inhibitor dacomitinib suppressed tumor growth. Although this model does not reflect all GBM tumors, it demonstrates that GBM’s heterogeneity, together with possible heterodimers between ERBB proteins, must be considered when designing targeted therapeutic strategies.

Additionally, ERBB4 localization on hyper-proliferative endothelial structures in GBM samples and on endothelial cells derived from GBM patient samples further supports the inclusion of ERBB4 in targeted therapeutic strategies for GBM. Although ERBB4 has been found in normal heart and human umbilical cord endothelium, and has been suggested to play a role in heregulin-induced angiogenesis [48], it has not previously been identified in tumor endothelium or on pericytes in GBM. Thus, ERBB4 has an angiogenic role in GBM, further highlighting the need to include an ERBB4 inhibitor in GBM therapy.

Overall, our study identified elevated expression of the oncogenic ERBB4 variant JM-a–CYT-2 in GBM and found p-ERBB4 to be an EGFR-independent prognostic marker for GBM. Additionally, we identified an angiogenic role for ERBB4 in GBM. Our data reveal ERBB4 as a rational therapeutic target in GBM and have important implications for the design of trials targeting the EGFR pathway, given that EGFR and ERBB4 co-expression may attenuate the response to EGFR-directed therapy.

## 4. Materials and Methods

### 4.1. Patient Samples

Fifty-three archived GBM samples, from patients who had undergone a diagnostic biopsy during 2007–2010 (mean age, 66.4 ± 2.2 years (range, 21–86); 66% male), were processed for pathological and immunohistochemistry (IHC) analysis, and matching clinical data were obtained. For RNA analysis, 28 GBM samples, plus 15 additional GBM samples with matching contralateral non-neoplastic normal brain (NNB) samples, were collected at autopsy and snap-frozen. Ten NNB samples were also collected at autopsy from donors with no known neurological condition or neuropathological findings (59.0 ± 2.3 years (48–72); 80% male). The study was approved by the Southern Health Human Ethics Committee (project 09023B), and the samples were from biobanks and institutions as in the Acknowledgments.

### 4.2. Cell Lines, Chemicals and Drugs

Various GBM cell lines and other cancer cell lines (as controls) were screened for ERBB4 expression by RT-qPCR (Table S1). SF767 GBM cells were from the Brain Tumor Research Center (UCSF, San Francisco, CA, USA); U87MG GBM cells, which are ERBB4 negative, were from ATCC (Manassas, VA, USA). ERBB4^E317K^ and U87ERBB4 cell line construction and culture conditions were as in the Supplementary Information. All cell lines were maintained in DMEM/F12 medium containing 5% FBS, 2 mM GlutaMAX and penicillin/streptomycin (Life Technologies, Mulgrave, Victoria, Australia) at 37 °C in a humidified 5% CO_2_ incubator. In addition, U87ERBB4 and ERBB4^E317K^ cells were maintained in 2 µg/mL puromycin (Life Technologies). Panitumumab was provided by Amgen (Sydney, Australia) and dacomitinib by Pfizer (Sydney, Australia), and heregulin-1β was from R&D Systems (Minneapolis, MN, USA).

### 4.3. Derivation of U87ERBB4 and U87ERBB4^E317K^ Cell Lines

A full-length cDNA clone encoding ERBB4, pDNR-Her4, was obtained from the PlasmID Database, Harvard University. PCR was used to amplify the insert cDNA for subcloning into the retroviral vector pBABE-puro [49]. Site-directed mutagenesis was used to introduce the E317K amino acid substitution (to generate the constitutively active form of ERBB4 identified in melanoma [28], U87ERBB4^E317K^). See the Supplementary Information for details.

### 4.4. RT-qPCR

RNA was extracted from snap-frozen GBM (*n* = 28) and NNB (*n* = 10) samples, plus contralaterally paired normal brain (paired NNB) (*n* = 15) and GBM (*n* = 15) samples, using TRIzol (Life Technologies) and was cleared of DNA with the RQ1 RNase-Free DNase kit as per the manufacturer’s instructions (Promega, Madison, WI, USA). FirstChoice Human Brain Reference RNA (Ref Brain) was from Ambion (Life Technologies). RT-qPCR conditions were as per the Supplementary Information.

### 4.5. Immunohistochemistry (IHC)

IHC was performed on 4-μm formalin-fixed paraffin-embedded (FFPE) serial sections of tumor samples as described in the Supplementary Information. Sections with no staining were classified as ‘negative’. Sections that were weakly stained with less than 50% of tumor cells stained were classified as ‘low’. Sections that were intensely stained with more than 50% of the tumor cells stained were classified as ‘high’. No sections were observed with weak staining of a large proportion of cells or intense staining of a small proportion of cells.

### 4.6. MTS and Bio-Plex Assays

Cells were processed according to protocols detailed in the Supplementary Information. Briefly, for MTS assays, CellTiter 96 Aqueous MTS solution (20 µL/well; Promega) was added to 6-day cell cultures, and the absorbance (490 nm) was measured, after 3 h at 37 °C, with a FLUOstar Optima plate reader (BMG Labtech, Offenburg, Germany). For Bio-Plex, lysates were analyzed for phospho-ERK (p-ERK) and p-AKT levels using Bio-Plex Phosphoprotein Detection Assays (Bio-Rad Laboratories, Hercules, CA, USA), according to the manufacturer’s protocol, and the Bio-Rad Bio-Plex 200 System with Bio-Plex Manager 5.0 software (Bio-Rad Laboratories, Hercules, CA, USA).

### 4.7. Gene Expression Profiling of ERBB Proteins in GBM-Derived Endothelial Cells

Brain tumor specimens were collected at MSKCC according to an IRB-approved protocol. GBM-derived endothelial cells from three GBM patients were isolated as previously described [50]. Immortalized human cerebral microvessel endothelial cells (HCMEC) (provided by B. Weksler, Weill Cornell Medical College, New York, NY, USA) were used as the positive control brain endothelial cells [51]. Detailed protocol in the Supplementary Information.

### 4.8. Xenograft Studies

Six-week-old female, athymic BALB/c mice were injected with cells and treated as per the Supplementary Information, in accordance with The Australian Code of Practice for the Care and Use of Animals for Scientific Purposes 2004 and as approved by MMC Ethics Committee A (approval 2011/76 on 6 November 2011).

### 4.9. TGCA Analysis

*ERBB4* gene expression data were collated from the first 100 samples of the unc.edu_GBM.AgilentG4502A_07_2 GBM Level 3 TCGA data set. Data were then arranged in ascending order in Microsoft Excel, and a heat map was applied. *ERBB4* isoform data were collated from the first 52 samples of the UNC__IlluminaHiSeq_RNASeqV2 Level 3 TCGA data set. Data were analyzed using Student’s *t*-test as described in Section 4.10. 

### 4.10. Statistical Analysis

Student’s *t*-test and one-way and two-way ANOVAs were used as indicated. Kaplan–Meier survival analysis was performed using the Gehan–Breslow–Wilcoxon test. Differences between groups were considered significant when *p* < 0.05. All tests were performed using GraphPad Prism v6.02 (La Jolla, CA, USA).

## 5. Conclusions

The oncogenic ERBB4 variant, JM-a–CYT-2, showed elevated expression in GBM compared to other variants. Furthermore, activated ERBB4, as measured by its phosphorylation, was an independent prognostic marker for GBM with these patients having a poorer prognosis. Finally, we showed that ERBB4 has a central role in GBM angiogenesis. Taken together, our data identifies ERBB4 as an oncogenic driver in GBM and therefore is a rational therapeutic target, an observation supported by our xenograft studies. Given that ERBB4 downstream signalling could attenuate drugs targeting the EGFR pathway, future trials should consider drugs that target both EGFR and ERBB4 to maximize clinical benefit in GBM. 

## Supplementary Materials

The following are available online at http://www.mdpi.com/2072-6694/10/8/243/s1, Table S1: Endogenous levels of ERBB4 in cancer cell lines. Figure S1: *ERBB4* and *ERBB4* variant expression profiles from TCGA data. A, Heat map of the relative expression of *ERBB4* mRNA in GBM sourced from the first 100 samples in the TCGA RNA SeqV2 dataset. High *ERBB4* expression was identified in 15% of patient samples (*n* = 100). B, Relative mRNA expression of *ERBB4* variants from GBM samples sourced from TCGA RNA SeqV2 dataset (*n* = 52) (* *p* < 0.0001). Figure S2: ERBB4 variant expression. Expression levels were determined in 28 GBM, 10 NNB and reference brain (Ref Brain) samples by RT-qPCR, and variant levels were standardized to total *ERBB4* mRNA levels. All measurements were made simultaneously but are displayed separately to aid comparison. ERBB4 JM-c (A), JM-d (B), CYT-1 (C) and CYT-2 (D) mRNA expression in NNB, GBM and Ref Brain samples. (* *p* < 0.05; Student’s *t*-test). All experiments were performed with three technical replicates. Figure S3: Serial sections comparing p-ERBB4 and p-EGFR staining intensity and localization in GBM. p-ERBB4 staining (DAB, brown) (A and C) was compared with the intensity and location of p-EGFR staining (DAB, brown) (B and D). If the stains co-localized (A and B), the area was removed from p-ERBB4 analysis. If p-ERBB4 and p-EGFR did not co-localize (C and D), the area was included in the analysis. This example is considered p-ERBB4^hi^/p-EGFR^neg^. Asterisks identify areas of comparison. Scale bars, 100 µm. Figure S4: Cell signaling changes following constitutive ERBB4 expression. p-ERK (A) and p-AKT (B) expression are shown normalized to total protein expression (signal ratio), as determined by Bio-Plex phospho-protein assay, in U87ERBB4 and U87ERBB4^E317K^ cells. Bars indicate mean values (*n* = 3). (* *p* < 0.05; Student’s *t*-test). All experiments were performed in triplicate. Figure S5: Deregulation of cell signaling in response to panitumumab. SF767 cells treated with panitumumab for 24 h exhibited an increase in the p-ERK signal ratio (*p* = 0.85) (A) and a minimal change in the p-AKT signal ratio (*p* = 0.34) (B), as determined by Bio-Plex phosphorylated protein assay, following treatment with 10 µg/mL panitumumab in vitro for 24 h. Furthermore, the increase in p-ERK protein was confirmed by western blotting (C). The signal ratio denotes the amount of phosphorylated protein divided by the total amount of protein. Bars indicate mean values (*n* = 2). All experiments were performed in duplicate. Figure S6: SF767 cells are inhibited by an ErbB4 antagonist. Growth of SF767 was analyzed in real time by exCELLigence. Addition of the ErbB4-Fc ligand trap at 48 h (dotted line) impeded the growth of SF767 cells. Figure S7: Expression of ERBB4, p-ERBB4, heregulin-1β and EGFR on vessels of GBM. IHC staining of GBM vessels with ERBB4 (A), p-ERBB4 (B), heregulin-1β (C) and EGFR (D). The ERBB protein was localized with DAB (brown), and the nuclei were counterstained with hematoxylin (blue). Scale bars, 25 µm. Figure S8: Serial sections of vascular ERBB4 protein expression. IHC double staining of endothelial cells with CD31 (VECTOR blue, blue) and ERBB4 (DAB, brown) (A,B) or smooth muscle actin (SMA) (DAB, brown) (C,D) in subcutaneous tissue (A,C) and U87-CM alginate plugs (B,D). Arrows identify pericytes (brown); asterisks identify endothelial cells. Scale bars, 25 µm. Results are representative of the results obtained for the four sample groups. Figure S9: Representative IHC images of blood vessels in xenografts shown in Figure 6. A, Samples were stained for CD31 (VECTOR Blue, blue) and smooth muscle actin (DAB, brown). Arrows indicate vessels. Scale bars, 50 µm. B, Samples were stained for CD31 (VECTOR Blue, blue), and nuclei were counterstained with Ki67 (VECTOR Red, red). Arrows indicate vessels. Scale bars, 50 µm.
