# Supplementary material for: Activation of ERBB4 in Glioblastoma Can Contribute to Increased Tumorigenicity and Influence Therapeutic Response
Source: Cancers (Basel). 2018 Jul 25;10(8):243. doi: 10.3390/cancers10080243 (PMC6116191; doi:10.3390/cancers10080243)
Supplement: Supplementary file 1 [file cancers-10-00243-s001.pdf]

SupplementaryMaterials

Activation of ERBB4 in Glioblastoma Can Contribute to Increased Tumorigenicity and Influence Therapeutic Response

Jacqueline F. Donoghue, Lauren T. Kerr, Naomi W. Alexander, Sameer A. Greenall, Anthony B. Longano, Nicholas G. Gottardo, Rong Wang, Viviane Tabar, Timothy E. Adams, Paul S. Mischel and Terrance G. Johns

1. **Detailed methods**

1.1 Immunohistochemistry

FFPE tissue sections were dewaxed, rehydrated and microwaved in citrate buffer for antigen retrieval. Following blocking of endogenous peroxidase for 15 minutes (in 3% H_2O_2) and blocking of non-specific protein binding for 1 h (with 10% goat serum (Sigma-Aldrich, Melbourne, Australia) in 0.1% BSA in PBS), samples were probed overnight (16 h) at 4 °C with primary antibodies specific for the following proteins; EGFR [rabbit anti human/mouse clone SC03; 1 µg/mL; Santa Cruz Biotech, Santa Cruz, CA, USA), ERBB4 (mouse anti human clone HFR-1; 1 µg/mL; Neomarkers, Fremont, CA, USA), p-EGFR (rabbit anti hu/mouse clone Y1086; 1 µg/mL; Life Technologies, Mulgrave, Australia), p-ERBB4 (rabbit anti hu/mouse clone Y1056); 1 µg/mL; Santa Cruz) and Ki67 (rabbit anti hu/mouse clone D3B5; 1 µg/mL; Cell Signaling Technology, Danvers, MA, USA). Following washing in TBST (Tris-buffered saline containing Tween 20), samples were incubated with biotinylated goat anti-rabbit or biotinylated goat anti-mouse secondary antibodies (Life Technologies) (1:200) for 1 h. A signal amplifier, ABC-HRP (Vector Laboratories, Burlingame, CA, CA), was subsequently added for 30 minutes, according to the manufacturer's instructions, followed by the chromogen DAB (Sigma-Aldrich) or VECTOR Red (Vector Laboratories) for 5 min. Slides were counterstained with hematoxylin, dehydrated and mounted.

Two investigators (A.B.L. and J.F.D.) were blinded to the samples and assessed the staining independently in accordance with a pre-established scoring chart. Sections with no staining were classified as 'negative'. Sections that were weakly stained with less than 50% of tumor cells stained were classified as 'low'. Sections that were intensely stained with more than 50% of the tumor cells stained were classified as 'high'. No sections were observed with weak staining of a large proportion of cells or intense staining of a small proportion of cells.

For vascular characterization, double stains were established: combinations of antibodies against the following proteins were incubated with prepared samples overnight (16 h): for human samples: ERBB4 (rabbit anti hu/mouse clone SC283; 1 µg/mL; Santa Cruz) plus either CD31 (mouse anti human clone JC70A; 1 µg/mL; Dako, Sydney, Australia) or smooth muscle actin (SMA) (mouse anti human clone 1A4; 1 µg/mL; Dako). For mouse samples CD31 (rat anti mouse clone SZ31; 0.5 µg/mL; Dianova, Hamburg, Germany) plus either ERBB4 (rabbit anti hu/mouse clone SC283; 1 µg/mL; Santa Cruz) or SMA (rabbit anti hu/mouse clone ab5694; 0.5 µg/mL; Abcam, Melbourne, Australia).

Following incubation, the samples were washed several times with TBST and biotinylated secondary antibodies were applied. Firstly, ERBB4 and SMA (rabbit) was incubated with biotinylated goat anti-rabbit antibody (1 h; 1:200) followed by streptavidin-HRP (1 h; 1:200) and colored with DAB chromogen for 5 min. After washing, CD31 and SMA (mouse) were incubated with biotinylated goat anti-mouse (1 h; 1:200) while CD31 (rat) was incubated with biotinylated rabbit anti rat (1 h; 1:200) followed anti-streptavidin alkaline phosphatase (Life Technologies) (1 h; 1:200) and colored with VECTOR Blue for 5 min (Vector Laboratories). All samples were washed with distilled water and mounted in aqueous mounting media (Dako Australia).

For microvessel counts, samples were scanned for areas of high vessel concentrations ('hotspots'), and four fields of view per sample were captured using a Leica microscope and capture unit using a 20x lens. Using ImageJ software, microvessels or proliferating cells were counted for each field of view, and the mean counts±SEM were recorded.

1.2 Derivation of U87ERBB4 and U87ERBB4^{E317K} Cell Lines

U87MG GBM cells were obtained from the ATCC. A full-length cDNA clone encoding ERBB4, pDNR-Her4, was obtained from the PlasmID Database, Harvard University. PCR was used to amplify the insert cDNA for subcloning into the retroviral vector pBABE-puro [1]. Site-directed mutagenesis was used to introduce the E317K amino acid substitution (to generate the constitutively active form of ERBB4 identified in melanoma [2], U87ERBB4^{E317K}), using the primer pair 5′-AGATGGAAGTAGAAAAAAATGGGATTAAA-3′ and 5′-TTTAATCCCATTTTTTTCTACTTCC ATCT-3′. The Plat-A amphotropic retrovirus packaging cell line was transfected with retroviral vectors corresponding to wild-type and mutant ERBB4 by using FuGENE (Roche, North Ryde, NSW, Australia). Virus-containing supernatants were then used to infect U87MG cells. Transfected cells were selected and maintained in the presence of puromycin (2 μg/mL).

1.3 MTS Proliferation Assay

Cells were seeded in triplicate at 1,000 cells/well in 96-well plates, allowed to adhere overnight, then washed in serum-free medium and incubated in medium containing 0.5% FBS at 37 °C, 5% CO_2 for 6 days. CellTiter 96 Aqueous MTS solution (20 μl/well; Promega, Madison, WI, USA) was added, and the absorbance (490 nm) was measured, after 3 h at 37 °C, with a FLUOstar Optima plate reader (BMG Labtech, Offenburg, Germany).

1.4 Bio-Plex Assay

Cells were seeded at 200,000 cells/well in six-well cell culture plates and left to attach overnight. Cells were washed with PBS and serum-free medium and then serum-starved in the presence of 10 μg/mL panitumumab overnight. Next, cells were washed and lyzed with Bio-Plex Cell Lysis buffer, and the lysates were analyzed for phospho-ERK (p-ERK) and p-AKT levels using Bio-Plex Phosphoprotein Detection Assays (Bio-Rad Laboratories, Hercules, CA, USA), according to the manufacturer's protocol, and the Bio-Rad Bio-Plex 200 System with Bio-Plex Manager 5.0 software.

1.5 xCELLigence Real-Time Analysis

The Real-Time Cell Analyzer (RTCA) SP instrument (Roche Diagnostics GmbH, North Ryde, NSW, Australia) was placed in a humidified incubator maintained at 37 °C with 5% CO_2. 2,500 SF767 cells were seeded in 96-well plates (E-plate 96, Roche Diagnostics GmbH) in media. Cells were initially monitored once every 2 min for 1 h and then once every hour. After addition of ErbB4-Fc, cells were monitored once every 10 min for 3 h and thereafter once every hour.

1.6 RT-qPCR Analysis

All RNA was converted to cDNA using SuperScript III (Sigma-Aldrich). The total ERBB4 and ERBB4 variant levels were analyzed by PCR with the 7900 FAST Real-Time PCR Thermocycler (Life Technologies). Thermocycling conditions were as follows: 95 °C for 10 min, followed by 40 cycles of 95 °C for 15 s and 60 °C for 60 s).

A generic human ERBB4 20x Probe/Primer pre-mix (Applied Biosystems; Life Technologies) was used for measuring total ERBB4 expression. ERBB4 variant primers and probes were designed based on allele-specific PCR, which relies upon the 3′ end of either the forward or reverse primer to discriminate between closely related mRNAs. The same reverse primer and probe was used for all ERBB4 variants, whereas different forward primers were used to distinguish ERBB4 variants [JM Forward Primers: JM-a: TCC ACT TTA CCA CAA CAT GCT AGA AC; JM-b: CAT CGG CCT GAT GGA TAG AAC; JM-c: CCA AAC TGC ACC CAA GGA A; JM-d: TCG GCC TGA TGG ATA GGT

GTA; JM Reverse Primer: AGA CCC ACA ATG ACC AGA ATG AA; JM Probe: 6FAM CCC ACC AAT TAC TCC MGBNFQ]; [CYT Forward Primers: CYT-1: ACA CCC CCA TGT CAG GAA AC; CYT-2: CAA GAA TTG ACT CGA ATA GGA ACC A; CYT Reverse Primer: ACA CTC CTT GTT CAG CAA AA; CYT Probe: 6FAM CCT CCA TCT CGG TAT AC MGBNFQ]. The sequences used to design primers and probes were sourced from NCBI NM_0052352 Human ERBB4 JM-a–CYT-1 Human and NM_001042599 Human JM-a–CYT-2. Efficiency of amplification for each primer set was validated using a 2-fold dilution series of cDNA. Efficiency ranged from 98.7–99.9%.

RT-qPCR was performed by amplifying triplicate samples containing 10-μL reaction mixture consisting of 2× gene expression mastermix (Applied Biosystems), 18 μM primer, 5 μM probe and 5 μg DNA. The H6PD gene was chosen as reference standard for normalization using the comparative Ct method [2-ΔΔCt] and analyzed using SDS 2.3 software (Applied Biosystems).

1.7 Gene Expression Profiling of ERBB Proteins in GBM-derived Endothelial Cells

Briefly, dissociated GBM cells were blocked with human FcR (1:20, Miltenyi Biotec, Bergisch Gladbach, Germany) at 4 °C for 20 min before a 30-min incubation with FITC–CD105 (1:40; BD Bioscience, North Ryde, NSW, Australia). CD105-positive cells (endothelial cells) and CD105-negative cells (non-endothelial cells) were then sorted by flow cytometry, using negative staining for DAPI (Sigma Aldrich, St. Louis, MO) as an indicator of live cells. Sorted GBM endothelial cells from two patients were cultured in M199 medium supplemented with 20 ng/mL VEGFA (Upstate Biotechnology, Lake Placid, NY, USA) or 20 ng/mL PDGF (R&D Systems, Minneapolis, MN, USA) as for 5 days. Total RNA was extracted with the RNeasy Kit (QIAGEN, Chadstone, VIC, Australia) and pre-treated with DNase (QIAGEN). Total RNA from six samples from three patients plus one HCMEC sample was hybridized to human gene expression arrays (Illumina) at Sloan Kettering Cancer Center's Genomics Core Facility, according to the manufacturer's instructions. The data were normalized by RMA algorithms. The heat map of ERBB expression was constructed with Microsoft Excel.

1.8 Xenografts

Mice were injected subcutaneously in each flank with $1×10^6$ SF767 cells or U87MG cells retrovirally transduced with U87ERBB4 or U87ERBB4^{E317K}. Tumors were measured twice per week with digital calipers, and volumes calculated as width^2 × 0.5 length. Once the average tumor volume per group reached 100 mm^3, 1 mg/mL panitumumab was administered intraperitoneally on alternate days for 14 days. Dacomitinib (Pfizer, West Ryde, NSW, Australia), a pan-ERBB inhibitor, was administered intraperitoneally (10 mg/kg in captisol) daily for 10 days. Once tumor volumes reached 1,000 mm^3, mice were euthanized and tumor collected for analysis. Tumor volumes are presented as mean volume ± SEM.

Alginate plugs were generated as previously described [3]. Briefly, 500 μl of 1.5% sodium alginate (Sigma-Aldrich) dissolved in sterile saline was layered on top of a 5mL volume of 80 mM calcium carbonate (Sigma-Aldrich) followed by incubation at 37 °C for 30 minutes. Set plugs were then washed in sterile water and stored at 4 °C. Plugs were then injected with either 100 μl of PBS or serum free conditioned media collected from U87MG cells over three days (U87-CM) and concentrated 5 times using an Amicon ultrafuge-15 (Merck-Millipore, Australia). BALB/c mice were anesthetized with ketamine (10 mg/mL) and xylazine (5 mg/mL), and an alginate plug was placed subcutaneously into both flanks. Mice then received daily injections of PBS (vehicle) or dacomitinib (10 mg/kg) for 7 days. Grafts were then collected and fixed in 10% buffered formalin for processing and IHC.

Supplementary Table and figures

Table S1. Endogenous levels of ERBB4 in cancer cell lines.

**Cell line**	**Total ERBB4**	**JM-a**	**JM-b**	**JM-c**	**JM-d**	**CYT-1**	**CYT-2**
GBM cell lines							
SF767	+++	++++	++	+	-	+	++
U87MG	-	-	-	-	-	-	-
GBML-1	-	-	-	-	-	-	-
GBML-2	-	-	-	-	-	-	-
GBM-4	-	-	-	-	-	-	-
GBM-6	-	-	-	-	-	-	-
CSC 020	-	-	-	-	-	-	-
Breast cancer cell lines							
MCF7	+++	+++	+	+	-	++	++
T-47D	++++	++++	++	+	-	++++	++

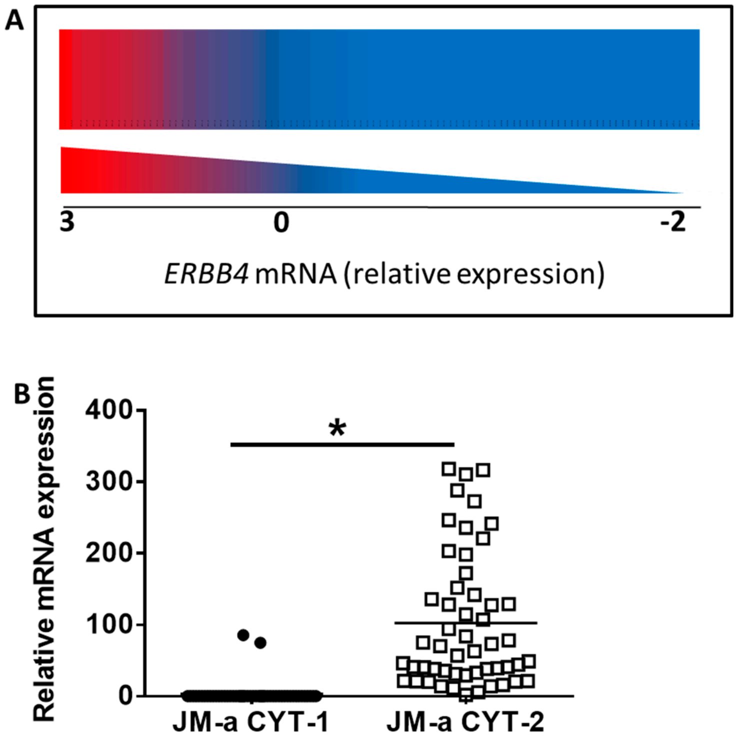

Figure S1. *ERBB4* and *ERBB4* variant expression profiles from TCGA data. **A**, Heat map of the relative expression of *ERBB4* mRNA in GBM sourced from the first 100 samples in the TCGA RNA SeqV2 dataset. High *ERBB4* expression was identified in 15% of patient samples ($n = 100$). **B**, Relative mRNA expression of *ERBB4* variants from GBM samples sourced from TCGA RNA SeqV2 dataset ($n = 52$) (* $p < 0.0001$).

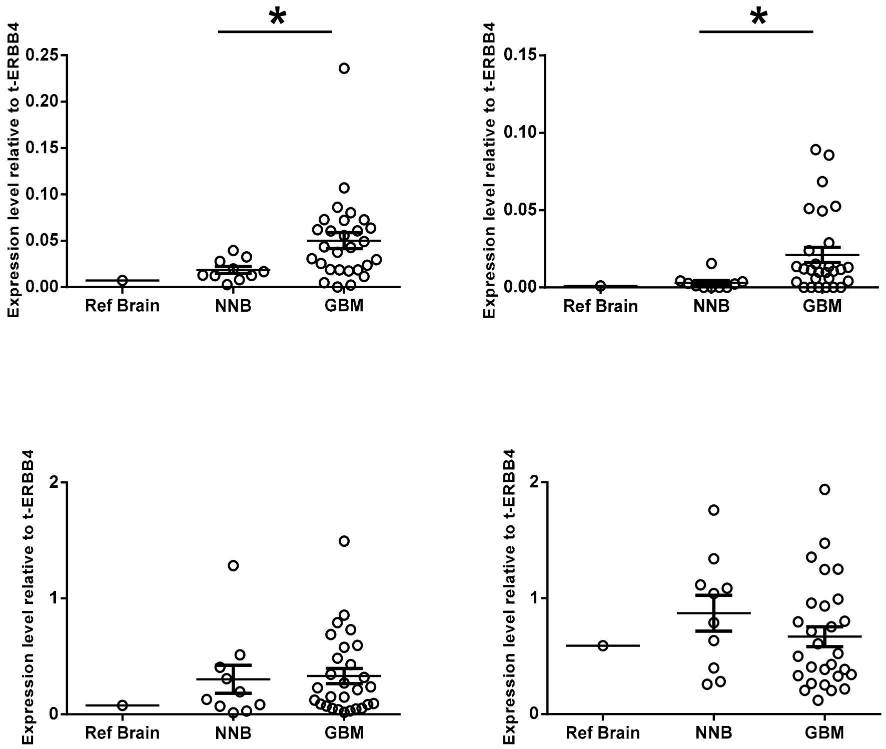

Figure S2. ERBB4 variant expression. Expression levels were determined in 28 GBM, 10 NNB and reference brain (Ref Brain) samples by RT-qPCR, and variant levels were standardized to total *ERBB4* mRNA levels. All measurements were made simultaneously but are displayed separately to aid comparison. ERBB4 JM-c (**A**), JM-d (**B**), CYT-1 (**C**) and CYT-2 (**D**) mRNA expression in NNB, GBM and Ref Brain samples. (* $p < 0.05$; Student's *t*-test). All experiments were performed with three technical replicates.

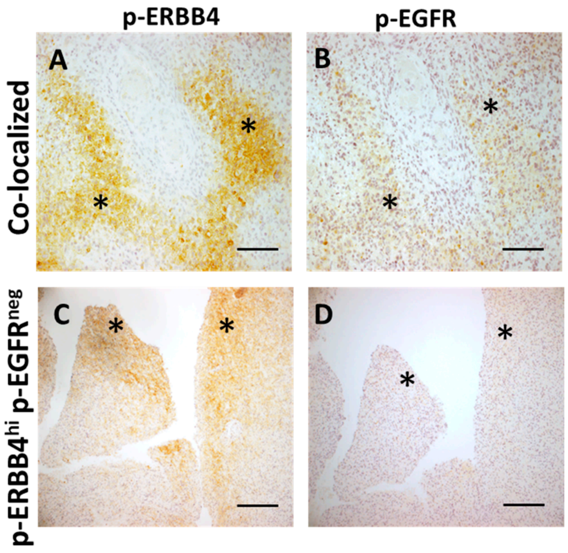

Figure S3. Serial sections comparing p-ERBB4 and p-EGFR staining intensity and localization in GBM. p-ERBB4 staining (3,3′-Diaminobenzidine (DAB), brown) (**A** and **C**) was compared with the intensity and location of p-EGFR staining (DAB, brown) (**B** and **D**). If the stains co-localized (**A** and **B**), the area was removed from p-ERBB4 analysis. If p-ERBB4 and p-EGFR did not co-localize (**C** and **D**), the area was included in the analysis. This example is considered p-ERBB4^{hi}/p-EGFR^{neg}. Asterisks identify areas of comparison. Scale bars, 100 µm.

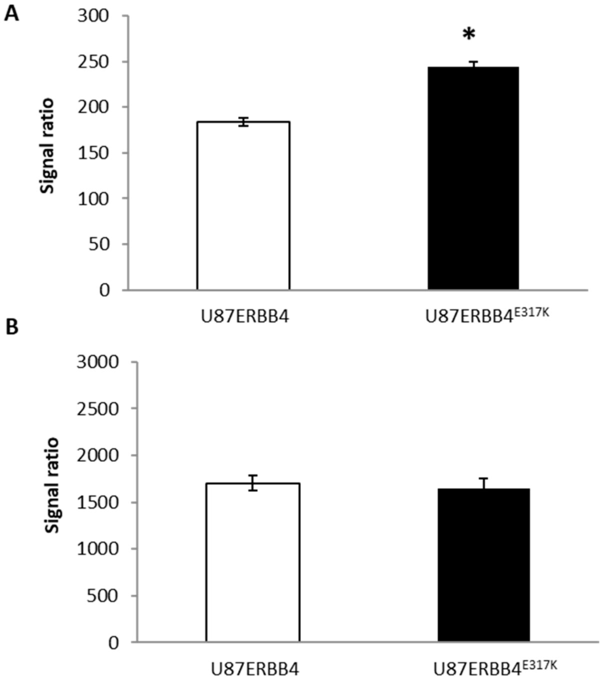

Figure S4. Cell signaling changes following constitutive ERBB4 expression**.** p-ERK (**A**) and p-AKT (**B**) expression are shown normalized to total protein expression (signal ratio), as determined by Bio-Plex phospho-protein assay, in U87ERBB4 and U87ERBB4^{E317K} cells. Bars indicate mean values (*n* = 3). (* $p < 0.05$; Student's *t*-test). All experiments were performed in triplicate.

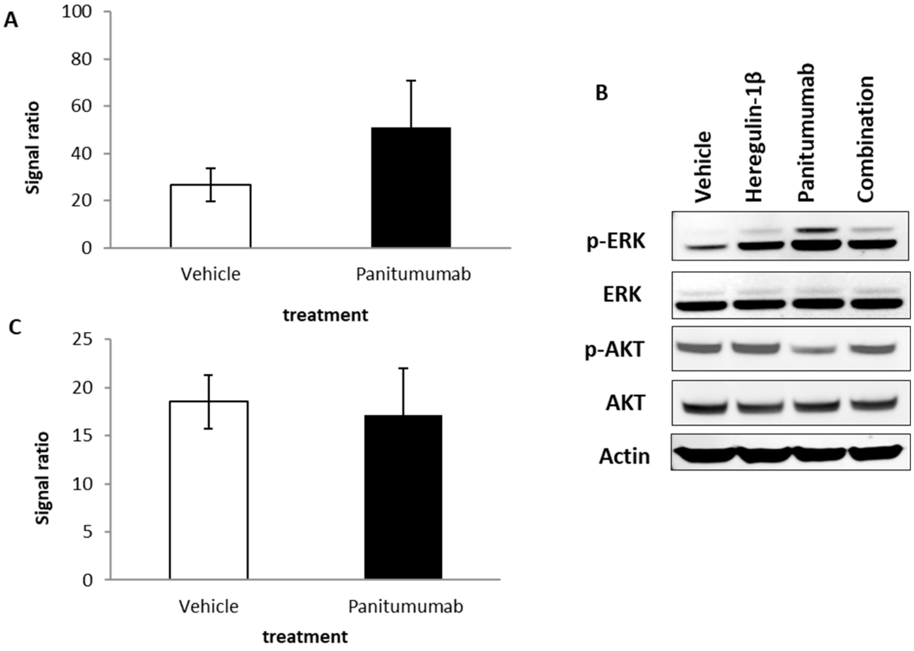

Figure S5. Deregulation of cell signaling in response to panitumumab. SF767 cells treated with panitumumab for 24 h exhibited an increase in the p-ERK signal ratio ($p = 0.85$) (**A**) and a minimal change in the p-AKT signal ratio ($p = 0.34$) (**B**), as determined by Bio-Plex phosphorylated protein assay, following treatment with 10 μg/mL panitumumab *in vitro* for 24 h. Furthermore, the increase in p-ERK protein was confirmed by western blotting (**C**). The signal ratio denotes the amount of phosphorylated protein divided by the total amount of protein. Bars indicate mean values (*n* = 2). All experiments were performed in duplicate.

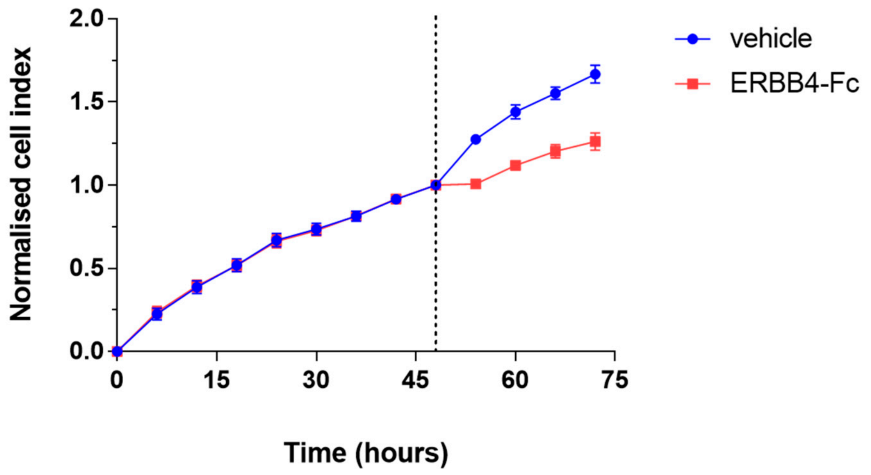

Figure S6. SF767 cells are inhibited by an ErbB4 antagonist. Growth of SF767 was analyzed in real time by exCELLigence. Addition of the ErbB4-Fc ligand trap at 48 h (dotted line) impeded the growth of SF767 cells.

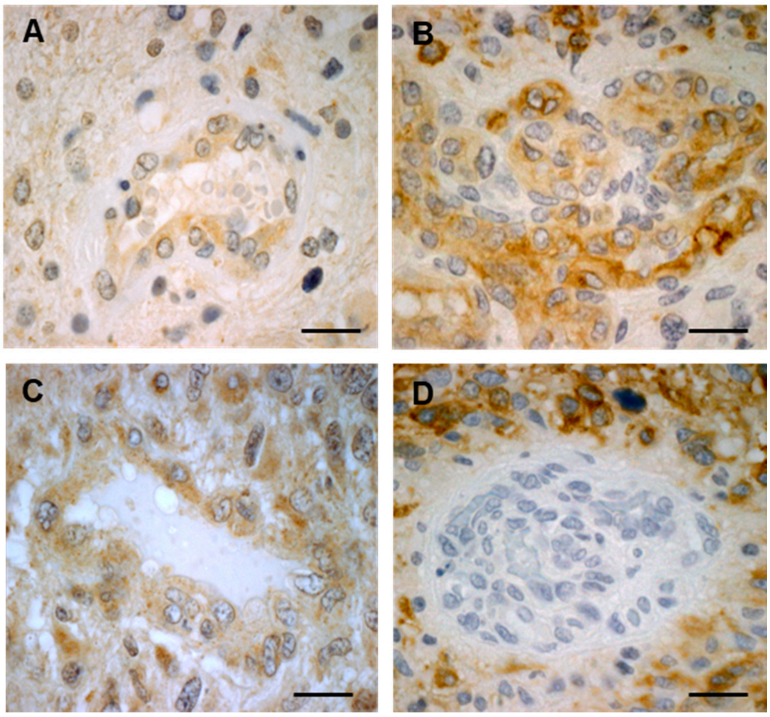

Figure S7. Expression of ERBB4, p-ERBB4, heregulin-1β and EGFR on vessels of GBM**.** IHC staining of GBM vessels with ERBB4 (**A**), p-ERBB4 (**B**), heregulin-1β (**C**) and EGFR (**D**). The ERBB protein was localized with DAB (brown), and the nuclei were counterstained with hematoxylin (blue). Scale bars, 25 μm.

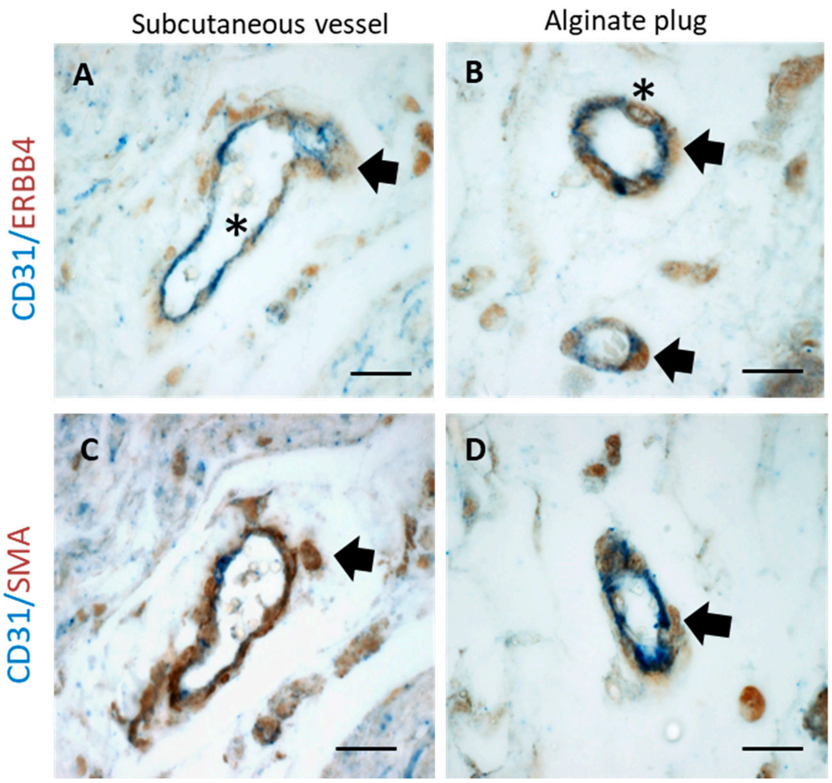

Figure S8. Serial sections of vascular ERBB4 protein expression. IHC double staining of endothelial cells with CD31 (VECTOR blue, blue) and ERBB4 (DAB, brown) (**A**,**B**) or smooth muscle actin (SMA) (DAB, brown) (**C**,**D**) in subcutaneous tissue (**A**,**C**) and U87-CM alginate plugs (**B**,**D**). Arrows identify pericytes (brown); asterisks identify endothelial cells. Scale bars, 25 µm. Results are representative of the results obtained for the four sample groups.

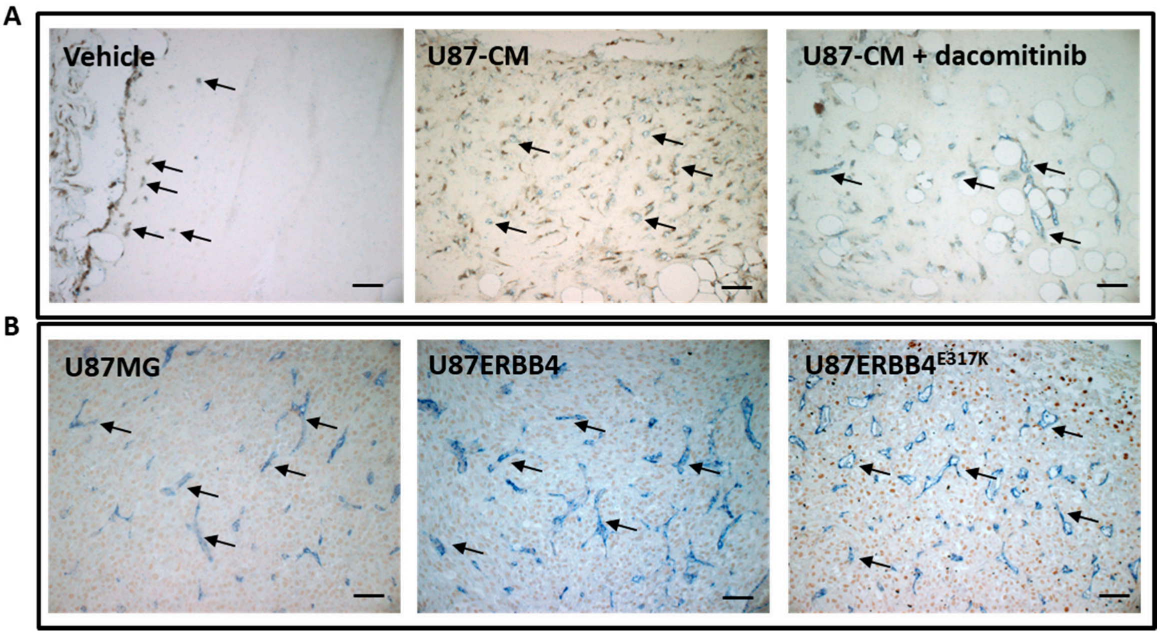

Figure S9. Representative IHC images of blood vessels in xenografts shown in Figure. 6. **A**, Samples were stained for CD31 (VECTOR Blue, blue) and smooth muscle actin (DAB, brown). Arrows indicate vessels. Scale bars, 50 µm. **B**, Samples were stained for CD31 (VECTOR Blue, blue), and nuclei were counterstained with Ki67 (VECTOR Red, red). Arrows indicate vessels. Scale bars, 50 µm.

References

1. Morgenstern, J.P.; Land, H. Advanced mammalian gene transfer: High titre retroviral vectors with multiple drug selection markers and a complementary helper-free packaging cell line. *Nucleic Acids Res.* **1990**, *18*, 3587–3596.
2. Prickett, T.D.; Agrawal, N.S.; Wei, X.; Yates, K.E.; Lin, J.C.; Wunderlich, J.R.; Cronin, J.C.; Cruz, P.; Rosenberg, S.A.; Samuels, Y. Analysis of the tyrosine kinome in melanoma reveals recurrent mutations in ERBB4. *Nat. Genet.* **2009**, *41*, 1127–1132.
3. Hoffmann, J.; Schirner, M.; Menrad, A.; Schneider, M.R. A highly sensitive model for quantification of in vivo tumor angiogenesis induced by alginate-encapsulated tumor cells. *Cancer Res.* **1997**, *57*, 3847–3851.

© 2018 by the authors. Licensee MDPI, Basel, Switzerland. This article is an open access article distributed under the terms and conditions of the Creative Commons Attribution (CC BY) license (http://creativecommons.org/licenses/by/4.0/).
